# How Cargo Identity Alters the Uptake of Cell-Penetrating Peptide (CPP)/Cargo Complexes: A Study on the Effect of Net Cargo Charge and Length

**DOI:** 10.3390/cells11071195

**Published:** 2022-04-01

**Authors:** Hannah C. Hymel, Alireza Rahnama, Olivia M. Sanchez, Dong Liu, Ted J. Gauthier, Adam T. Melvin

**Affiliations:** 1 Cain Department of Chemical Engineering, Louisiana State University, Baton Rouge, LA 70803, USA; hhymel5@lsu.edu (H.C.H.); ralire1@lsu.edu (A.R.); osanch2@lsu.edu (O.M.S.); 2 LSU AgCenter Biotechnology Laboratory, Louisiana State University, Baton Rouge, LA 70803, USA; doliu@agcenter.lsu.edu (D.L.); tgauthier@agcenter.lsu.edu (T.J.G.)

**Keywords:** cell penetrating peptide, intracellular delivery, cargo

## Abstract

Cell-penetrating peptides (CPPs) have emerged as a powerful tool for the delivery of otherwise impermeable cargoes into intact cells. Recent efforts to improve the delivery capability of peptides have mainly focused on the identity of the CPP; however, there is evidence that the identity of the cargo itself affects the uptake. The goal of this work was to investigate how the characteristics of a peptide cargo, including net charge and length, either enhance or diminish the internalization efficiency of the CPP/cargo complex. A small library of CPP/cargo complexes were synthesized consisting of structured and unstructured CPPs with cargoes of net positive, negative, or neutral charge and lengths of 4 or 8 amino acids. Cargoes with a net positive charge were found to enhance the overall uptake of the complexes while net neutral and negatively charged cargoes diminished uptake. Conversely, the net length of the cargo had no significant effect on uptake of the CPP/cargo complexes. Microcopy images confirmed the increased uptake of the positively charged cargoes; however, an increase in punctate regions with the addition of a cargo was also observed. The effects of the net positively charged cargoes were confirmed with both structured and unstructured CPPs, which demonstrated similar trends of an increase in uptake with the addition of positively charged residues. These findings demonstrate that the net charge of cargoes impacts the uptake of the complex, which can be considered in the future when designing peptide-based reporters or therapeutics.

## 1. Introduction

The cellular plasma membrane is vital for cell survival and function, but it presents a major challenge for the intracellular delivery of therapeutic agents or biosensors. The plasma membrane selectively excludes hydrophilic, negatively charged, or large molecules, with several existing delivery approaches (e.g., electroporation or microinjection) being limited in their ability to deliver the cargo without damaging the cell or altering its behavior [1,2,3]. Cell-penetrating peptides (CPPs) have been developed for the passive delivery of impermeable molecules without altering cellular function. CPPs are most commonly less than 30 amino acids in length and can penetrate the plasma membrane without compromising cellular integrity to deliver an otherwise impermeable cargo to intracellular compartments [4,5]. These peptides have successfully delivered various molecular cargoes, such as nanoparticles, proteins, peptides, and DNA [6,7,8,9]. The first CPP with permeability properties was described more than 30 years ago and was derived from the transactivator of transcription (HIV-TAT) protein [10,11]. Shortly after, the homeodomain of Antennapedia, a Hox gene found in Drosophila, was also shown to be capable of entering intact cells [12]. Since then, numerous CPPs have been discovered of both natural and synthetic origin. Examples of truncated versions of these proteins include penetratin (from Antennapedia) and the 13-residue sequence TAT_48–60_ (derived from HIV-TAT) [13,14]. While the structure and conformation of CPPs are variable, they are often characterized by a significant number of positively charged or hydrophobic residues that interact with the plasma membrane to facilitate penetration into the cell [15]. Amphipathic CPPs with alternating regions of hydrophilic and hydrophobic amino acids in their sequence have also been discovered to enter cells with examples including Pep-1and pVEC [16,17]. While numerous CPPs have been identified and shown to penetrate the plasma membrane, one limitation associated with numerous CPPs is poor intracellular stability, resulting in rapid degradation by peptidases and proteases. Several strategies have been explored to address this weakness, including the incorporation of protease-resistant non-natural amino acids [18] or by modifying the primary structure such as the cyclization of linear peptides and the inclusion of peptide stapling [19,20,21]. Another approach to increase intracellular stabilities is to utilize CPPs with a distinct secondary structure to prevent entry into the catalytic cleft of proteases and peptidases. For example, peptides incorporating a rigid d-Pro-Gly β-turn to create a secondary structure demonstrated increased stability compared to unstructured peptides [22]. Safa et al. previously characterized the cell permeability characteristics of these previously identified “protectides” with a β-hairpin secondary structure, including the peptide RWRWR (RWVRVpGOWIRQ) [23]. The study showed that although the overall uptake levels of the β-hairpin CPPs are lower compared to unstructured CPPs such as nona-arginine, they exhibit enhanced cytosolic stability and protease resistance, demonstrating a tradeoff between net uptake into cells and the lifetime of the peptide within the intact cells.

In addition to improving the permeability and stability of the CPPs themselves, there is significant interest in utilizing known CPPs to deliver bioactive cargoes, such as nanoparticles, therapeutics, or biosensors, to cells. CPPs have found great success of late as delivery vehicles for peptide-based therapeutics, such as PROTACs (proteolysis targeting chimeras), which facilitate the degradation of “undruggable” proteins by selectively ubiquitinating the target protein for proteasome-mediated degradation [24,25]. CPPs have also been incorporated into peptide-based biosensors, where they are conjugated to a cargo consisting of a short, enzyme-specific, recognition or binding sequence for a target protein and an analytical handle (e.g., fluorescent marker) capable of directly quantifying protein activity [26]. Recently, Safa et al. developed a cell-permeable biosensor to detect deubiquitinating enzyme (DUB) activity in intact cancer cells [27]. The reporter incorporated the previously established RWRWR β-hairpin CPP with a short DUB-binding peptide sequence (LRGG), and a fluorescent molecule (AFC). They observed that the conjugation of the four amino acid binding sequence diminished the net uptake of the peptide-based reporter compared to the uptake of the RWRWR CPP itself. RWRWR uptake in HeLa cells was found to occur rapidly (<10 min); however, the RWRWR-LRGG-AFC reporter required >90 min to be sufficiently internalized. This suggests that addition of a cargo affects the uptake efficiency of the CPP and, thus, should be considered when designing CPP-conjugated therapeutics or biosensors. Prior work investigating the uptake of CPP/cargo complexes have mostly focused on how the identity (e.g., the sequence and structure) of the CPP alters cargo uptake by conjugating the same cargo onto a variety of established CPPs [28,29]. Patel et al. conjugated green fluorescent protein (GFP) as a model cargo onto seven different CPPs, such as penetratin, octa-arginine, TAT, Transportan, and Xentry. The study demonstrated that uptake of the cargo was not only dependent on the CPP, it was conjugated too, but also on the cell line it was tested in, including HeLa, HEK, and HepG2 [30]. Khayyatnejad et al. utilized a hybrid cyclic-linear CPP, ((RW)_5_)K(RW)_X_, to transport two different cargoes: a phosphopeptide and an anti-HIV drug [31]. While the goal of this study was to determine how the addition of positive and hydrophobic residues to the linear chain of the CPP affected uptake, it also demonstrated there was a significantly different uptake of these two different CPP/cargo complexes. The CPP–phosphopeptide complex showed higher cellular uptake than the CPP–drug complex, presumably due to the negatively charged phosphopeptide interacting more efficiently with the positively charged CPP when physically mixed to form the CPP/cargo complex. However, there was no further investigation into this inconsistency. Despite mounting evidence that the conjugation of a cargo affects the uptake of the CPP, there are still relatively few studies on how varying the characteristics of the cargo itself influences the internalization of the CPP/cargo complexes.

The goal of this work is to provide insight on how the identity of a cargo affects the uptake efficiency of the CPP/cargo complex into intact cells. To accomplish this, a library of peptides was created incorporating short peptide cargoes of varying net length and net charge conjugated to two different CPPs: (1) the structured β-hairpin RWRWR CPP; and (2) the commonly used unstructured nona-arginine CPP (RRRRRRRRR) [23]. Fluorometry studies demonstrated that positively charged cargoes increased overall peptide uptake when compared to the cargo-less CPP, while the conjugation of neutral and negatively charged cargoes diminished overall peptide uptake, sometimes completely halting cellular internalization. Conversely, there was no significant difference in the uptake of CPP/cargo complexes with the same net cargo charge but varying net cargo length. It was also observed that changing the location of positive residues within the cargo sequence, but not altering the net cargo charge, did not affect the overall uptake of the CPP/cargo complex. Interestingly, these trends were observed in both the structured and unstructured CPPs, suggesting that cargo identity is just as important as CPP identity when designing CPP-conjugated therapeutics or biosensors. Thus, the net charge but not the net length of the cargo will enhance or diminish the uptake of CPP/cargo complexes.

## 2. Materials and Methods

### 2.1. Chemicals and Reagents

Fmoc-protected amino acids, 2-(6-Chloro-1H-benzotriazole-1-yl)-1, -1,3,3-tetramethylaminium hexafluorophosphate (HCTU), trifluoroacetic acid (TFA), and rink amide SS resin, were purchased from Advance ChemTech, Louisville, KY, USA. Nα-Fmoc-Nδ-allyloxycabonyl-L-ornithine (Fmoc-Orn[Aloc]-OH) was purchased from Chem-Impex International, Inc., Wood Dale, IL, USA. 1-Hydroxy-6-(trifluoromethyl) benzotriazole (HOBt) and (Ethyl cyano[hydroxyimino]acetato)-tri-(1-pyrrolidinyl)-phosphonium (PyBOP) were purchased from Novabiochem, Billerica, MA, USA. Dimethylformamide (DMF) was purchased from Protein Technologies, Tucson, AZ, USA. Diisopropylethylamine (DIEA), triisopropylsilane (TIPS), tetrakis(triphenylphosphine) palladium (0) (palladium), 5(6)-carboxyfluorescein (FAM), chloroform (CHCl_3_), methanol (MeOH), dichloromethane (DCM), and N-methylmorpholine (NMM) were purchased from Sigma-Aldrich, St. Louis, MO, USA. Glacial acetic acid was purchased from Alfa Aesar, Ward Hill, MA, USA. Acetic anhydride was purchased from Fisher Scientific, Fair Lawn, NJ, USA. Commercial peptide ARG (FAM-RRRRRRRRR) was purchased from AnaSpec, Fremont, CA, USA. All the salts used for the preparation of assay buffers were purchased from Sigma Aldrich, St. Louis, MO, USA.

### 2.2. Peptide Synthesis and Purification

Peptides were synthesized as previously described by Safa et al. [23]. All peptides were synthesized on a Tribute peptide synthesizer (Protein Technologies, Tucson, AZ, USA) using a standard Fmoc peptide chemistry protocol on a 100 μmol scale using rink amide resin (189 mg, 0.53 mmol/g). Fivefold excess of Fmoc-amino acids (Fmoc-Arg(Pbf)-OH, Fmoc-Trp(Boc)-OH, Fmoc-Val-OH, Fmoc-Orn-OH, Fmoc-d-Pro-OH, Fmoc-Gly-OH, Fmoc-Ile-OH, Fmoc-Gln(Trt)-OH, Fmoc-Orn(Aloc)-OH, and HCTU) in the presence of 10 equivalents of NMM were used for each of the amino acid coupling steps (10 min) with N-methyl-2-pyrrolidone (NMP) as the solvent. A solution of acetic anhydride, NMM, and NMP (1:1:3) was added to the deprotected resin and shaken for 30 min to acetylate the N terminus of the peptide. Once the peptide synthesis was complete, the resin was washed with DMF (3 × 30 s) and then DCM (3 × 30 s). The Aloc group was removed with 3-fold excess of palladium in 4 mL of CHCl_3_-HOAc-NMM (37:2:1) under nitrogen for 2 h. The resin was then washed with DCM (3 × 30 s) followed by DMF (3 × 30 s). In the dark, FAM was coupled to the delta nitrogen of the ornithine side chain with 4-fold excess of FAM, HOBt, PyBOP, and DIEA in 3 mL DMF for 24 h and then repeated for 8 h. The peptide resin was washed with DMF (3 × 30 s) and DCM (3 × 30 s). The peptide was cleaved from the resin and sidechain deprotected using TFA/water/TIPS (4 mL, 95:2.5:2.5) for 3 h and collected in a 50-mL centrifuge tube. The cleavage reaction was repeated for 10 min. The cleavage solutions for the peptide were combined and concentrated in vacuo. Cold diethyl ether was then added to the peptide solution to precipitate the crude peptide. The peptide was centrifuged for 10 min (4000 rpm) and the ether layer decanted. Fresh cold diethyl ether was added, and the pelleted peptide was resuspended. The peptide was centrifuged again, and the procedure was repeated 5 times in total. After the final ether wash, the peptide pellet was dissolved in 5 mL water containing 0.1% TFA, frozen, and lyophilized.

High-performance liquid chromatography (HPLC) analysis was performed with a Waters 616 pump, Waters 2707 Autosampler, and 996 Photodiode Assay Detector which were controlled by Waters (Milford, MA, USA) Empower 2 software. The separation was performed on an Agilent (Santa Clara, CA, USA) Zorbax 300SB-C18 (5 μm, 4.6 × 250 mm) with an Agilent guard column Zorbax 300SB-C18 (5 μm, 4.6 × 12.5 mm). Elution was done with a linear 5–55% gradient of solvent B (0.1% TFA in acetonitrile) into A (0.1% TFA in water) over 50 min at a 1 mL/min flow rate with UV detection at 442 nm. Preparative HPLC runs were performed with a Waters prep LC Controller, Waters Sample Injector, and 2489 UV/Visible Detector which are controlled by Waters (Milford, MA, USA) Empower 2 software. The separation was performed on an Agilent Zorbax 300SBC18 PrepHT column (7 μm, 21.2 × 250 mm) with Zorbax 300SB-C18 PrepHT guard column (7 μm, 21.2 × 10 mm) using a linear 5–55% gradient of solvent B (0.1% TFA in acetonitrile) into A (0.1% TFA in water) over 50 min at a 20 mL/min flow rate with UV detection at 215 nm. Fractions of high (>95%) HPLC purity of each peptide and with the expected mass were combined and lyophilized. HPLC purification and mass spectrometry validation of the RWRWR peptide has been previously reported [23]. Representative data for the CPP/cargo complexes with both positive and negative cargoes conjugated to the RWRWR CPP as well as a positive cargo conjugated to the nona-arginine CPP are presented in Figures S1–S3.

### 2.3. Circular Dichroism

Peptide concentrations were determined based on the absorbance of FAM at 492 nm. Circular dichroism (CD) spectroscopy data were collected as previously described by Safa et al. [23] using a J-815 CD spectrometer (JASCO, Easton, MD, USA). Spectra were generated at 25 °C with a wavelength scan (260–185 nm) using a 50 nm/min scanning speed in a 0.1 cm cell. Data pitch and accumulation were 1 and 3 nm, respectively. The scan mode was continuous. The smoothing method was Savitzky–Golay with a convolution width of 7. All peptides were at a final concentration of 40 μM in a 10 mM sodium phosphate buffer.

### 2.4. Cell Culture and Lysate Generation

HeLa cells (LSU AgCenter Tissue Culture Facility) were maintained in Dulbecco’s modified eagle medium (DMEM, Corning Inc., Corning, NY, USA) supplemented with 10% *v*/*v* HyClone Cosmic Calf Serum (VWR Life Sciences Seradigm, Radnor, PA, USA). HeLa lysates were generated by detaching cells with trypsin, resuspending in media, and pelleting the cells. The pellet was washed twice in phosphate buffered saline (PBS; 137 mM NaCl, 10 mM Na_2_HPO_4_, 27 mM KCl, and 1.75 mM KH_2_PO_4_ at pH 7.4). The pellet was then resuspended in an approximately equivalent volume of mammalian protein extraction reagent (ThermoFisher Scientific, Carlsbad, CA, USA) to the volume of the cell pellet, then vortexed for 10 min at 900 rcf. The samples were centrifuged at 14,000 rcf for 15 min at 4 °C. The supernatant was transferred to a new microcentrifuge tube and stored on ice until use. Total protein concentration was determined using a NanoDrop spectrophotometer (Thermo Scientific, Madison, WI, USA).

### 2.5. Quantification of CPP and CPP/Cargo Uptake

The quantification of peptide uptake was performed using a protocol previously utilized by Safa et al. [23] and Vaithiyanathan et al. [18]. Three days prior to the experiment, HeLa cells were seeded at a density of 1 × 10^4^ cells/mL in 12 well plates. Prior to experiments, peptides were reconstituted in sodium phosphate buffer (2.26 mM NaH_2_PO_4_·H_2_O and 8.43 mM Na_2_HPO_4_·7H_2_O) and the peptide concentration was determined using a NanoDrop at the wavelength of 492 nm using the UV-vis function. On the day of the experiment, peptides were diluted to the desired final concentration with extracellular buffer, ECB (5.036 mM HEPES pH 7.4, 136.89 mM NaCl, 2.68 mM KCl, 2.066 mM MgCl_2_·6H_2_O, 1.8 mM, CaCl_2_·2H_2_O, and 5.55 mM glucose). Cells were washed once with PBS followed by peptide incubation (500 μL/well) at 37 °C. Plates were wrapped in aluminum foil during incubation to avoid deactivating the FAM tag on the peptide. Following the incubation, the peptide solutions were removed, and cells were washed twice with ECB. Cells were then trypsinized (250 μL/well) for 10 min at room temperature. The cells were resuspended in ECB (1 mL/well) and transferred to microcentrifuge tubes. The samples were centrifuged at 1800 rcf for 2.5 min. The supernatant was removed, and the isolated cells were lysed with 0.1 M NaOH (250 μL/tube). Samples were centrifuged again at 14,000 rcf for 5 min to remove any remaining protein bound to the membranes before transferring 150 μL of clarified lysate to a 96 well plate. Fluorometry (Perkin Elmer (Waltham, MA, USA) Wallac 1420 VICTOR2 multilabel HTS counter) was used to quantify the signal resulting from the FAM tag using an excitation filter of 490 nm and an emission filter of 535 nm. For each experiment, a no peptide control (cells incubated with ECB only) was performed. To measure the background signal of each peptide, the peptide solutions used in each experiment were analyzed by fluorometry to normalize the fluorescent signal for each sample. Signals were normalized using Equation (1):

Normalized Fluorescence = 1000 × (F − C)/(P − B)(1)

where F denotes the fluorescent signal of cells incubated with peptide, C denotes the fluorescent signal of cells incubated with no peptide, P denotes the average fluorescent signal of the peptide in suspension, and B denotes the average fluorescent signal of the ECB. Reported data for each experiment is the average of triplicate samples. Statistical analysis was carried out using JMP Pro 16 software (SAS Institute).

### 2.6. Cell Viability Assay

Two days prior to the experiment, HeLa cells were plated at a density of 1 × 10^5^ cells/well in a 96 well plate. Cells were washed once with PBS and incubated with peptide solutions for 2 h at 37 °C. Following this incubation period, cells were washed with ECB and incubated with a mixture of 10 μL alamarBlue Viability Reagent (ThermoFisher Scientific, Carlsbad, CA, USA) in 90 μL serum-free media for 4 h at 37 °C. The fluorescent signal of alamarBlue was measured using fluorometry with an excitation filter of 532 nm and an emission filter of 535 nm. A no peptide control was also performed to normalize the fluorescent signals. Signals were normalized using Equation (2):

Normalized Cell Viability = F/C(2)

where F denotes the fluorescent signal of cells incubated with peptide and C denotes the fluorescent signal of cells incubated with no peptide. The reported data are the average of triplicate samples.

### 2.7. Fluorescent Microscopy

One day prior to the experiment, HeLa cells were seeded in 8-chambered Falcon Culture Slides (Corning) at a density of 3 × 10^4^ cells/mL. On the day of the experiment, cells were washed once with ECB before incubation with 10 μM peptide solution (500 μL/well) for 1 h at 37 °C. Following incubation, cells were washed twice with ECB then incubated with 8 μM Hoechst 3342 nuclei acid stain (ThermoFisher Scientific, Carlsbad, CA, USA) for 20 min at room temperature. Cellular fluorescence was visualized using a Leica DMi8 inverted microscope outfitted with a 40× objective (Leica N PLAN L, 0.55× correction). Images were acquired using the digital CMOS camera C11440 (Hamamatsu Photonics K.K., Japan) with a fixed exposure time of 500 ms for the FITC filter, 150 ms for the DAPI filter, and 20 ms for brightfield. The following excitation/emission filters (Chroma Tech. Corp, Bellows Falls, VT, USA) were used to image the device: fluorescein isothiocyanate-FITC (λ_ex_ 440–520 nm and λ_em_ 497–557 nm) for capturing the signal from FAM and DAPI (λ_ex_ 335–385 nm and λ_em_ 405–465 nm) for capturing the signal from the Hoescht stain. Image acquisition was controlled using the Leica Application Suite software (LAS X, version 3.7.4.23463), where all images were recorded using the same parameters. The imaging was followed by quantitative analysis, where a manual line scan region of interest (ROI) was drawn across the center of the cells to quantify their fluorescent signals. Random ROIs were also drawn in free space not containing cells to measure background noise for each image. The fluorescent values were normalized by subtracting the noise from the measured signal.

## 3. Results and Discussion

### 3.1. Net Positive Cargo Charge Enhances the Cellular Uptake of the CPP/Cargo Complex

**Figure 1:**
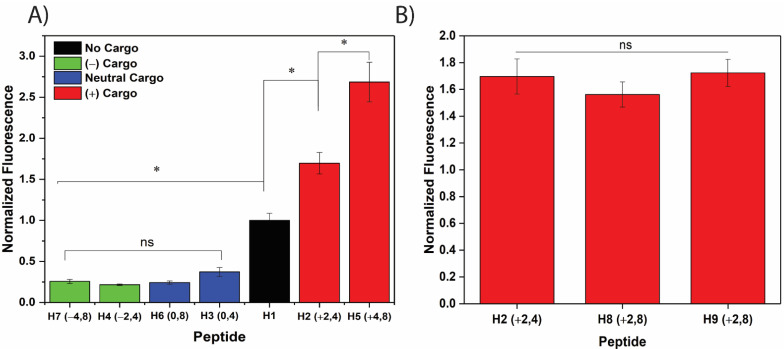
Investigation on the role of net charge and length on the internalization of CPP/cargo complexes. Intact cells were incubated with 10 μM of peptide solution for 1 h followed by lysis and quantification with fluorometry. (**A**) Cargoes of varying charge were conjugated to the H1 CPP to determine the effect of net cargo charge on peptide uptake. The data suggests that an increase in cargo charge results in a significant increase in peptide uptake, while neutral and negatively charged cargoes diminish uptake. (**B**) Cargoes of +2 charge but varying lengths were compared to determine the effect of cargo length on peptide uptake. The permeability of peptides with similar cargo charges but differing cargo length were not found to be statistically different. Additionally, the position of arginine residues was varied in peptides H8 and H9, with no significant effect on peptide internalization. * Denotes *p* < 0.0001. Experiments were conducted in triplicate to produce error bars.

To investigate the role of net cargo length and charge on the uptake efficiency of CPP/cargo complexes, a library of peptides (Table 1) was designed and synthesized by conjugating eight different cargoes to the RWRWR β-hairpin CPP (herein referred to as H1) and three cargoes to the nona-arginine CPP (herein referred to as R1). The cargoes consisted of different combinations of glycine (G, neutral charge), arginine (R, positive charge), and glutamic acid (E, negative charge), where manipulating the number of residues in the cargo sequence resulted in net cargo charges of −4, −2, 0, +2, and +4 and net cargo lengths of 4 and 8 residues. Peptides H2, H3, and H4 have a 4-residue cargo with net charges of +2, 0, and −2, respectively, conjugated to the H1 CPP. Peptides H5, H6, and H7 consist of an 8-residue cargo of +4, 0, and −4 net charge, respectively, attached to the H1 CPP. Finally, CPP/cargo complexes H8 and H9 both have an equal cargo length of 8 residues, and net cargo charge of +2, with different positioning of the positively charged arginine residues on the cargo sequence. CD spectra was used to confirm the addition of a cargo did not affect the overall structure of the peptide. H5, which has an 8-residue cargo with a net charge of +4, exhibited a minimum near 205 nm, which is characteristic of the β-hairpin and also observed in the H1 peptide (Figure S4). Although the effects of net charge on the CPP sequence have been abundantly studied, it is unknown how the electrostatic charge of the cargo conjugate would play a role on the CPP/cargo complex translocation [32,33]. Time-dependent analysis of peptide uptake in HeLa cells showed the H1 CPP and the H2 and H5 CPP/cargo complexes (with a net positive cargo charge) having the greatest penetration, with a single factor ANOVA revealing the uptake was time-dependent. The CPP/cargo complexes with a net neutral or net negative charge demonstrated much lower uptake, with H3 and H7 showing no time dependence (Figure S5 and Table S1). To better explore the role of cargo charge and length, one time point was isolated for further, in-depth examination. Cellular uptake studies were performed on the CPP by itself (H1) and the CPP/cargo complexes (H2–H7) at a concentration of 10 µM, incubation time of 60 min, and incubation temperature of 37 °C (Figure 1A). The uptake of the CPP/cargo complexes were normalized against the uptake of the H1 CPP to visualize how the role of cargo enhanced or diminished the uptake of the complex. It was found that the addition of cargoes with a net neutral or negative net charge (H3, H4, H6, and H7) diminished uptake while conjugation of cargoes with a net positive charge significantly enhanced internalization of the CPP/cargo complex when compared to the H1 CPP alone. A Fisher’s LSD test indicated that while there was no statistically significant difference in the uptake for negative and neutral cargoes, there was a significant difference between the H1 CPP and the CPP/cargo complexes with a net positive charge (Table S2). Additionally, increasing the number of arginine residues from 2 (H2) to 4 (H5) resulted in a 58% increase in CPP/cargo uptake. It has been shown that the plasma membrane of mammalian cells predominantly consists of glycerol-based phospholipids that result in negative electrostatic charge [34]. Moreover, it has been reported that increasing the presence of anionic phospholipids on the outer leaflet of cancer cells increase the negative charge on their plasma membrane [34,35]. These findings have been utilized in the design of CPPs to include positively charged amino acids, such as arginine, ornithine, or lysine, in the sequence to facilitate cellular translocation by electrostatic interaction with the negatively charged components on the plasma membrane. Futaki et al. highlighted the cellular translocation capabilities of arginine-rich peptides while suggesting that a common, yet unknown membrane penetration mechanism is observed for all such peptides [32]. Arginine has been favored over lysine when designing CPPs with positively charged amino acids with improved internalization in arginine-rich CPPs due the presence of a guanidium group [36,37,38]. While the benefits of incorporating positive residues on the CPP sequences is widely known, the presence of negative residues in the CPP/cargo complex can be beneficial as well. Conjugation of negatively charged macromolecular cargoes to positively charged CPPs has been shown to result in charge neutralization. This neutralization effect has been implemented in preventing non-specific CPP uptake in drug delivery applications [39,40].

The effect of net cargo length was then investigated after establishing that a net positive cargo charge enhanced CPP/cargo uptake. The uptake of three CPP/cargo complexes with a net charge of +2 and varying lengths (H2, H8, and H9) were compared (Figure 1B). A one-way ANOVA showed no significant difference in cellular uptake for the three CPP/cargo complexes, suggesting the net cargo length does not enhance or diminish peptide internalization (Table S3). Next, the effect of the location of the positively charged residues within the cargo was investigated to determine if amino acid placement impacted CPP/cargo uptake or if it was just due to the net positive charge. Studying the placement of the positively charged amino acids on the cargo sequence was motivated by prior efforts that have highlighted the orientational effects of a positive charge on the CPPs’ cellular penetration [41]. CPP/cargo complex H9 was similar in design to H8, with an overall net positive charge of +2 and length of 8 residues; however, in H8 the two arginine residues are placed closer to the C-terminus, whereas in H9 they are placed closer to the N-terminus. It was shown that the location of the positively charged arginine residues on the cargo sequence did not affect CPP/cargo complex cellular uptake, as no statistically significant difference was observed between peptides H8 (+2, 8) and H9 (+2, 8) (Figure 1B and Table S3). These results summarize the important of net cargo charge on the uptake of CPP/cargo complexes; however, the net length and the location of the positively charged residues do not enhance or diminish cellular penetration.

### 3.2. Entry of the CPP/Cargo Complex into Intact Cells Does Not Significantly Diminish Cellular Viability

**Figure 2:**
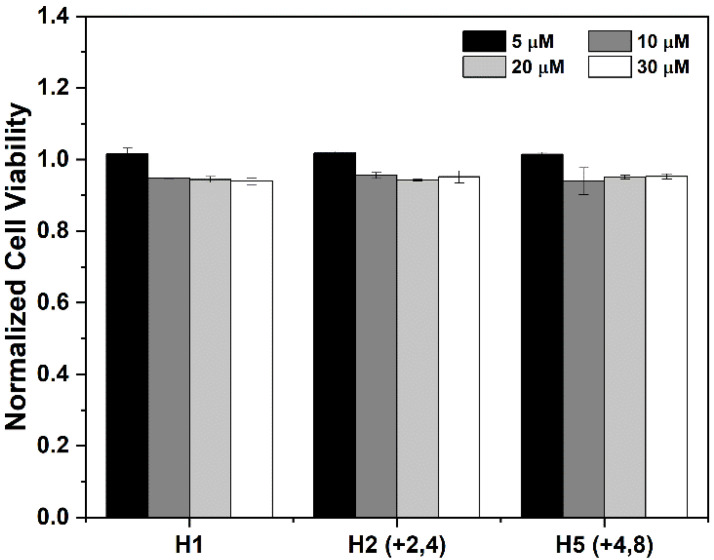
CPP/cargo complex uptake demonstrates minimal effect on cellular viability. Intact HeLa cells were incubated with the indicated concentrations of peptide solutions for 2 h at 37 °C. Cells were washed with ECB and incubated with alamarBlue stain for 4 h. Viability was quantified using fluorometry. Results show no significant change in viability between the peptides at each concentration, suggesting conjugation of cargo does not result in cytotoxicity. Experiments were conducted in triplicate to produce error bars.

With potential to be utilized in therapeutic applications such as drug delivery systems, cytotoxicity is a pivotal consideration when designing CPP/cargo complexes. Prior work suggested that the conjugation of a cargo to CPPs has the potential to alter the cytotoxicity of traditionally non-toxic CPPs. El-Andaloussi et al. compared the uptake and cytotoxicity of CPPs TAT and penetratin when conjugated to different cargo molecules, including dsDNA, streptavidin, and avidin, and speculated that the CPP/cargo complex toxicity depends on both CPP concentration and cargo molecule [42]. Cardozo et al. also observed a significant, cargo-length-dependent cytotoxicity when comparing CPP/cargo complexes, with peptide cargo sequences of 31 residues conjugated to TAT demonstrating toxic effects at 10 μM, 22 residue sequences demonstrating toxic effects at 30 μM, and 10 residues sequences appearing harmless up to 100 μM [43]. As such, the effect of a CPP/cargo complex on cellular viability was studied to expand upon these prior findings for the RWRWR CPPs. Three peptides with the highest observed uptake (H1 (CPP), H2 (+2, 4), and H5(+4, 8)) were incubated with HeLa cells for 4 h at 37 °C at four different concentrations to determine if the CPP itself or the CPP/cargo complexes diminished cellular viability (Figure 2). At 5 µM, all three peptides did not result in a decrease in cellular viability which is similar to previous studies that demonstrated minimal toxic effects at low concentrations [43]. Increasing the peptide concentration to 10, 20, and 30 μM slightly diminished cellular viability by ~5%, with no significant changes in cellular viability between the three doses. A single factor ANOVA revealed that peptide concentration did not significantly diminish cellular viability (Table S4), which suggests the conjugation of a cargo to the H1 CPP does not result in any cytotoxicity. The significance of exploring the dose-dependence of a peptide’s cytotoxicity lies in the potential in vivo applications, as by knowing the effects of CPP-mediated delivery of therapeutics on cellular viability, optimal decisions can be made depending on the applied concentrations.

### 3.3. CPP/Cargo Complexes Exhibited a Non-Uniform Fluorescence Distribution in Intact HeLa Cells

**Figure 3:**
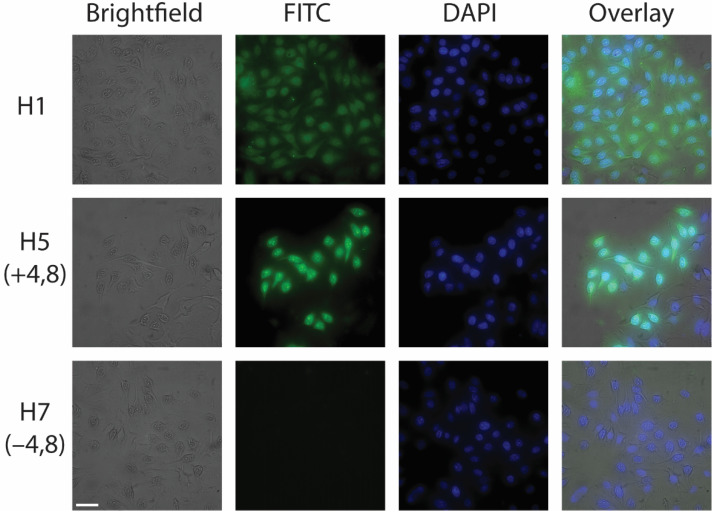
Visualization of the intracellular distribution of peptides in HeLa cells. A total of 10 μM peptide solutions were incubated with cells seeded on glass imaging chambers for 60 min at 37 °C. Cells were washed with ECB and incubated with 8 μM Hoechst stain for 20 min prior to imaging. Representative images include brightfield, FITC (for peptide uptake), and DAPI (Hoechst) filters. Scale bar is 50 μm. Images demonstrate increased uptake with the addition of positive residues in the cargo (H5) and diminished uptake with the addition of negative residues (H7).

**Figure 4:**
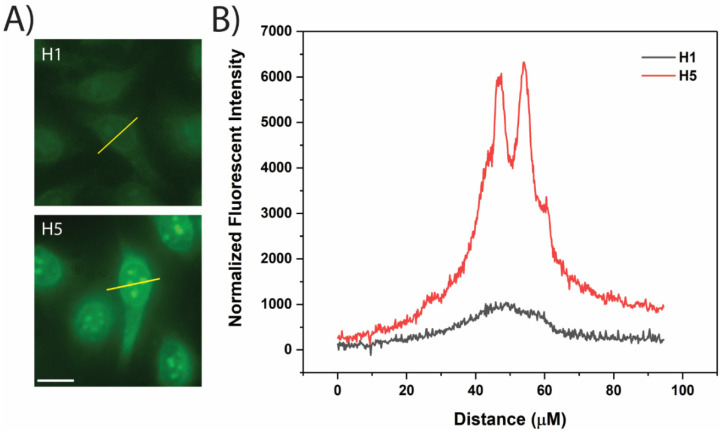
Analysis of peptide intracellular distribution. (**A**) Line scans shown in yellow were drawn across individual cells to demonstrate the fluorescent distribution of H1 and H5. Scale bar is 20 μm. (**B**) Plots of line scan data show a smooth curve for H1 while H5 resulted in sharp peaks, suggesting the addition of a cargo may lead to endosomal entrapment.

Live-cell fluorescent microscopy was performed to both validate cellular uptake and provide insight into the intracellular distribution of the peptides within the intact cells. One major challenge associated with select CPPs is that they can be retained within endosomes after cellular uptake, substantially limiting their ability to deliver biomolecules to the cytosol. Fluorescent microscopy is a useful tool to visualize punctate regions within the cell resulting from endosomal entrapment [44]. HeLa cells were incubated with 10 μM H1 (CPP), H5 (+4, 8), and H7 (−4, 8) for 60 min at 37 °C prior to imaging. Similar to the fluorometry results in Figure 1A, the fluorescence microscopy images confirmed the successful cellular uptake of CPP (H1) and the CPP/cargo complex with a net positive cargo charge (H5) and impermeability of the CPP/cargo complex with a net negative cargo charge (H7) (Figure 3). While H1 appeared to be evenly distributed throughout the cell, punctate regions of increased fluorescence were observed in the cells incubated with H5. This was further confirmed by performing line scans across individual cells (Figure 4A). Unlike the mostly smooth, uniform line seen for H1, the normalized fluorescent intensity across a cell that has internalized H5 demonstrates multiple sharp peaks (Figure 4B). This suggests the addition of the positively charged cargo can result in a greater degree of endosomal entrapment. However, Deprey et al. note that both nuclear exclusion and nuclear enrichment could demonstrate punctate staining, as could the formation of peptide aggregates [44]. The heterogeneity that exists in cancer cells at both intracellular and intercellular levels can potentially explain the difference in uptake efficiency when analyzed at the single cell level [45,46]. This was previously studied when Safa et al. investigated the cell-to-cell heterogeneity of β-hairpin CPP permeability (including H1) and were able to provide a quantitative analysis of peptide uptake variability among individual cells [27]. A similar analysis was performed here measuring the average fluorescence intensity in individual cells from a small population incubated with the H1 and H5 peptide (Figure S6). The results display a substantially higher heterogeneity in the cells incubated with H5 when compared to cells incubated with H1, suggesting the addition of the cargo results a wider variation in peptide uptake efficiency in HeLa cells. Moreover, the microscopy data support the findings from the fluorometric studies (Figure 1A) that the CPP/cargo with a net positive charge (H5) exhibits a substantially higher average uptake than the CPP itself (H1). However, the images also show that a trade-off exists when conjugating a positively charged cargo. While H5 does exhibit enhanced uptake, there appears to be a poorer overall distribution within the cell, which could be due to entrapment within the endosomes.

### 3.4. Net Cargo Charge, but Not Net Cargo Length, Enhance CPP/Cargo Complex Uptake in Both Structured and Unstructured CPPs

**Figure 5:**
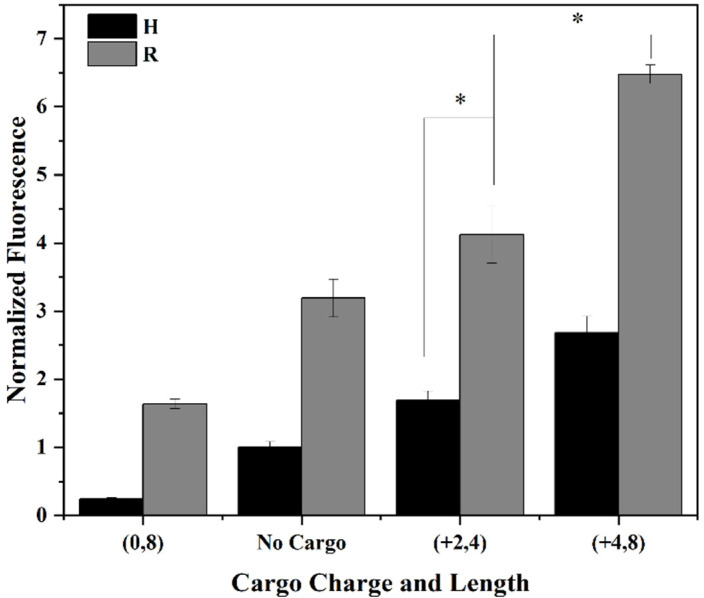
Comparison of cargo net charge and length effect on the uptake efficiency of unstructured (R) and structured (H) CPP. Cargoes of varying length and charge were attached to the β-hairpin (H) or poly-arginine (R) CPP. HeLa cells were incubated with 10 µM of peptide for 60 min, at 37 °C. Data suggest a similar trend to the effect on R1 CPP, as was observed with the H1 CPP, with the positively charged cargoes, (+2, 4) and (+4, 8), enhancing the uptake efficiency of the CPP/cargo complex on both R1 and H1 CPPs, while the neutrally charged cargo (0, 8) exhibits diminished uptake levels compared to that of the R1 and H1 CPPs alone. Experiments were conducted in triplicate to produce error bars. * Denotes *p* < 0.0001.

Prior work by Safa et al. demonstrated that the unstructured nona-arginine CPP exhibited 2-fold greater uptake efficiency, but the RWRWR (H1) CPP demonstrated a 20-fold increased intracellular stability [23]. With the majority of well characterized CPPs being unstructured, and considering the differences in uptake levels, cytotoxicity, and intracellular stability that has been observed when comparing unstructured and structured CPPs, it was important to compare the effects of net cargo charge and length on both unstructured (nona-arginine, R1) and structured (RWRWR, H1) CPPs. To examine the role of cargo on an unstructured CPP, two positively charged cargoes, R2 (+2, 4) and R3 (+4, 8), were conjugated to R1. Time-dependent analysis of peptide uptake in HeLa cells showed the R2 and R3 CPP/cargo complexes demonstrated greater uptake that the R1 CPP (Figure S7). A single-factor ANOVA revealed the uptake was time-dependent for both CPP/cargo complexes with a positively charged cargo (Table S6). Live-cell fluorescent microscopy was performed to visualize the intracellular distribution of the peptides. The images demonstrate that while there are punctate regions with the uptake of R1, the addition of a positively charged cargo appears to result in a greater degree of punctate regions throughout the cell (Figure S8). To compare the role of cargo on both structured and unstructured CPPs, an additional complex incorporating a neutral cargo on R1 was created. A similar trend was observed with the R1 CPP that was seen with the H1 CPP with positive net cargo charge enhancing the uptake of the CPP/cargo complex (Figure 5). The addition of a +2 net charge (R2) resulted in a 29% increase in uptake while the addition of a +4 net charge (R3) resulted in a 103% increase in uptake for the CPP/cargo complexes compared to the R1 CPP. Similar to what was shown for the H1 CPP, the conjugation of a neutrally charged cargo of 8 residues to the R1 CPP also led to a significant decrease in peptide uptake. The 50% decrease in cellular internalization of R4 (0, 8) compared to R1 CPP, confirms the negative effect of adding a neutrally charged cargo to unstructured CPP R1 uptake efficiency. Fisher’s LSD confirmed the statistical significance of the charge-dependent increase and decrease in uptake of peptides for both CPPs (Table S5). Moreover, overall higher permeability is observed from peptides with the R1 CPP in all four cases, compared to their counterparts with H1 CPP. Both R2 and R3 demonstrated a 140% increase compared to H2 and H5, respectively. This finding is in agreement with literature that have shown CPPs with a greater number of arginine residues provide increased cellular uptake [47]. Patel et al. studied the role of CPPs in effectively delivering cargo intracellularly [30]. The study used common CPPs, such as TAT and octa-arginine, as well as cyclic variations that had previously demonstrated enhanced uptake compared to their linear counterparts. When using GFP as a model cargo, it was observed that the cyclic CPP/cargo complexes maintained greater uptake compared to linear CPP/cargo complexes. The study presented here demonstrates cargoes of varying charges have similar effects on different CPPs. Moreover, the behavior of unstructured (R1) and structured (H1) CPPs was shown to remain the same when delivering different cargoes into the cytosol.

## 4. Conclusions

Previous studies demonstrated that the addition of a short peptide cargo to the RWRWR CPP diminished uptake of the CPP/cargo complex. Thus, this study aimed to further explore this observation by conjugating peptide cargoes of four and eight amino acids and varying the net charges onto the CPP to investigate the effect on uptake of the CPP/cargo complex. The cellular uptake of the CPP/cargo complexes was found to be greatly diminished when the cargo had a net neutral or negative charge; however, with a net positive charge, the uptake increased, with a 58% increase with the addition of two positive residues. A comparison of cargoes with +2 net charge of different lengths resulted in no significant difference in uptake, suggesting charge but not length were important factors in uptake of the complex. Additionally, the position of the arginine residues within the cargo did not affect the uptake of the complex. Viability studies showed that while there was a small drop in viability with an increase in peptide concentration from 5 to 10 µM, there was no significant difference between the CPP and CPP/cargo complexes at each tested concentration, suggesting the cargo is not toxic. Live-cell microscopy images visualized peptide localization in the cytosol for peptides H1 and H5, while the negatively charged cargo of H7 resulted in diminished uptake. Moreover, line scans of the microscopy images demonstrated that while the CPP, H1, was evenly distributed throughout the cell, the CPP/cargo complex, H5, resulted in punctate regions of fluorescence, suggesting there may be a tradeoff between enhanced uptake and greater endosomal entrapment. Finally, the effect of positively charged cargoes was compared for both structured and unstructured CPPs. As had been observed with the structured CPP, a neutral cargo on nona-arginine reduced uptake of the complex, while positively charged cargoes increased uptake. Cargoes conjugated to the unstructured CPP also demonstrated greater uptake than their respective cargoes on the structured CPP. These findings demonstrate that while the identity of the CPP is an important factor, the net charge of the cargo can also greatly enhance or dimmish uptake of the CPP/cargo complex, which can be useful considerations for developing future therapeutic or biosensing agents.

## Figures and Tables

**Table 1:** Library of CPP/cargo complexes to study the role cargo charge and length in uptake.

Name	Sequence	Cargo Charge	Cargo Length
H1	RWVRVpGO(FAM)WIRQ		
H2	RWVRVpGO(FAM)WIRQ-GRGR	+2	4-mer
H3	RWVRVpGO(FAM)WIRQ-GGGG	0	4-mer
H4	RWVRVpGO(FAM)WIRQ-GEGE	−2	4-mer
H5	RWVRVpGO(FAM)WIRQ-GRGRGRGR	+4	8-mer
H6	RWVRVpGO(FAM)WIRQ-GGGGGGGG	0	8-mer
H7	RWVRVpGO(FAM)WIRQ-GEGEGEGE	−4	8-mer
H8	RWVRVpGO(FAM)WIRQ-GGGGGRGR	+2	8-mer
H9	RWVRVpGO(FAM)WIRQ-GRGRGGGG	+2	8-mer
R1	FAM-RRRRRRRRR		
R2	FAM-RRRRRRRRR-GRGR	+2	4-mer
R3	FAM-RRRRRRRRR-GRGRGRGR	+4	8-mer
R4	FAM-RRRRRRRRR-GGGGGGGG	0	8-mer

Charge and length of cargoes are listed when applicable. Peptides utilizing the RWRWR β-hairpin CPP are denoted with an “H” in their name, while those utilizing the nona-arginine CPP are denoted with an “R”. FAM denotes 5,6 carboxyfluorescein and “p” denotes D-proline.

## Data Availability

The data that support the findings of this study are available within the article and its Supplementary Material.

## Supplementary Materials

The following are available online at https://www.mdpi.com/article/10.3390/cells11071195/s1, Figure S1: HPLC trace and mass spectrometry analysis (MALDI-TOF) of the H5 peptide, Figure S2: HPLC trace and mass spectrometry analysis (MALDI-TOF) of the H7 peptide, Figure S3: HPLC trace and mass spectrometry analysis (MALDI-TOF) of the R2 peptide, Figure S4: CD spectra of CPP/cargo complex, Figure S5: Structured CPP/cargo complexes with time-dependent internalization of peptides in HeLa cells, Figure S6: Comparison of fluorescent intensity of internalized peptides in HeLa cells, Figure S7: Unstructured CPP/cargo complexes with time-dependent internalization of peptides in HeLa cells, Figure S8: Visualization of intracellular distribution of unstructured CPP/cargo complexes in HeLa cells, Table S1: One-way ANOVA of the effect of time on CPP/cargo complex uptake, Table S2: Statistical comparison of net cargo charge on CPP/cargo complex uptake, Table S3: One-way ANOVA on of the effect of net cargo length on the internalization of CPP/cargo complexes, Table S4: One-way ANOVA of the effect of CPP/cargo complex uptake on HeLa cell viability, Table S5: Statistical comparison of net cargo charge on CPP/cargo complexes using both structured (H) and unstructured (R) CPPs, Table S6: One-way ANOVA of the effect of time on unstructured CPP/cargo complex uptake.
