# Supplementary material for: How Cargo Identity Alters the Uptake of Cell-Penetrating Peptide (CPP)/Cargo Complexes: A Study on the Effect of Net Cargo Charge and Length
Source: Cells. 2022 Apr 1;11(7):1195. doi: 10.3390/cells11071195 (PMC8997848; doi:10.3390/cells11071195)
Supplement: Supplementary file 1 [file cells-11-01195-s001.zip › cells-1655493-supplementary.pdf]

How cargo identity alters the uptake of cell penetrating peptide (CPP)/cargo complexes: A study on the effect of net cargo charge and length

Hannah C. Hymel[1#], Alireza Rahnama[1#], Olivia M. Sanchez[1], Ted J. Gauthier[2], and Adam T. Melvin[1]

[1]*Cain Department of Chemical Engineering, Louisiana State University, Baton Rouge, LA, 70803*
[2]*LSU AgCenter Biotechnology Lab, Louisiana State University, Baton Rouge, LA 70803*
[#]Equal Contribution

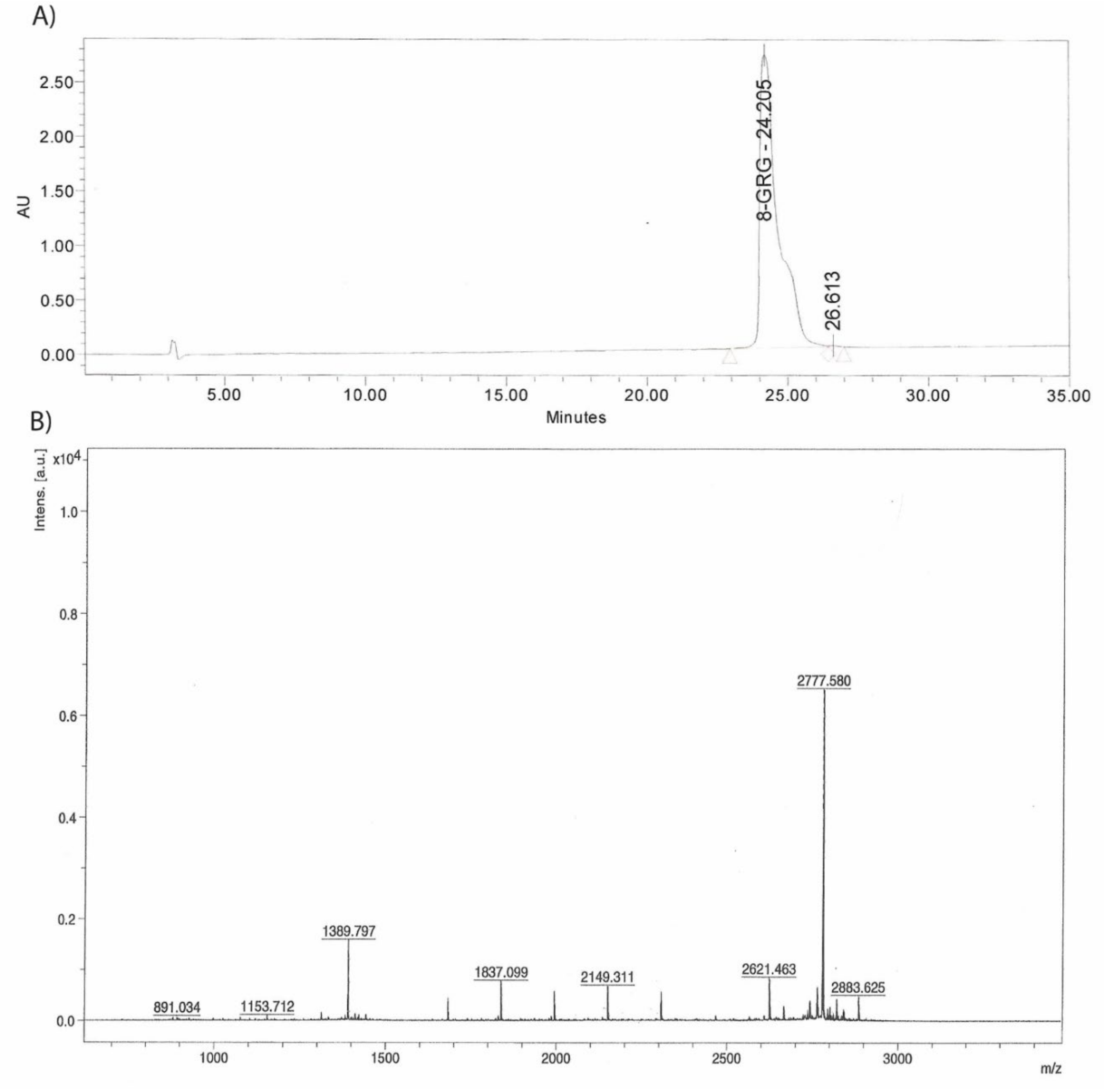

Figure S1. HPLC trace and mass spectrometry analysis (MALDI-TOF) of the H5 peptide. (A) HPLC separation was performed with a linear 5% to 55% gradient of solvent B (0.1% TFA in acetonitrile) into A (0.1% TFA in water) over 50 min at a 1 mL/min flow rate with UV detection at 442 nm. (B) MALDI-TOF of the peptide yielded an observed mass of 2777.580 Da ($[M+H]^+$)

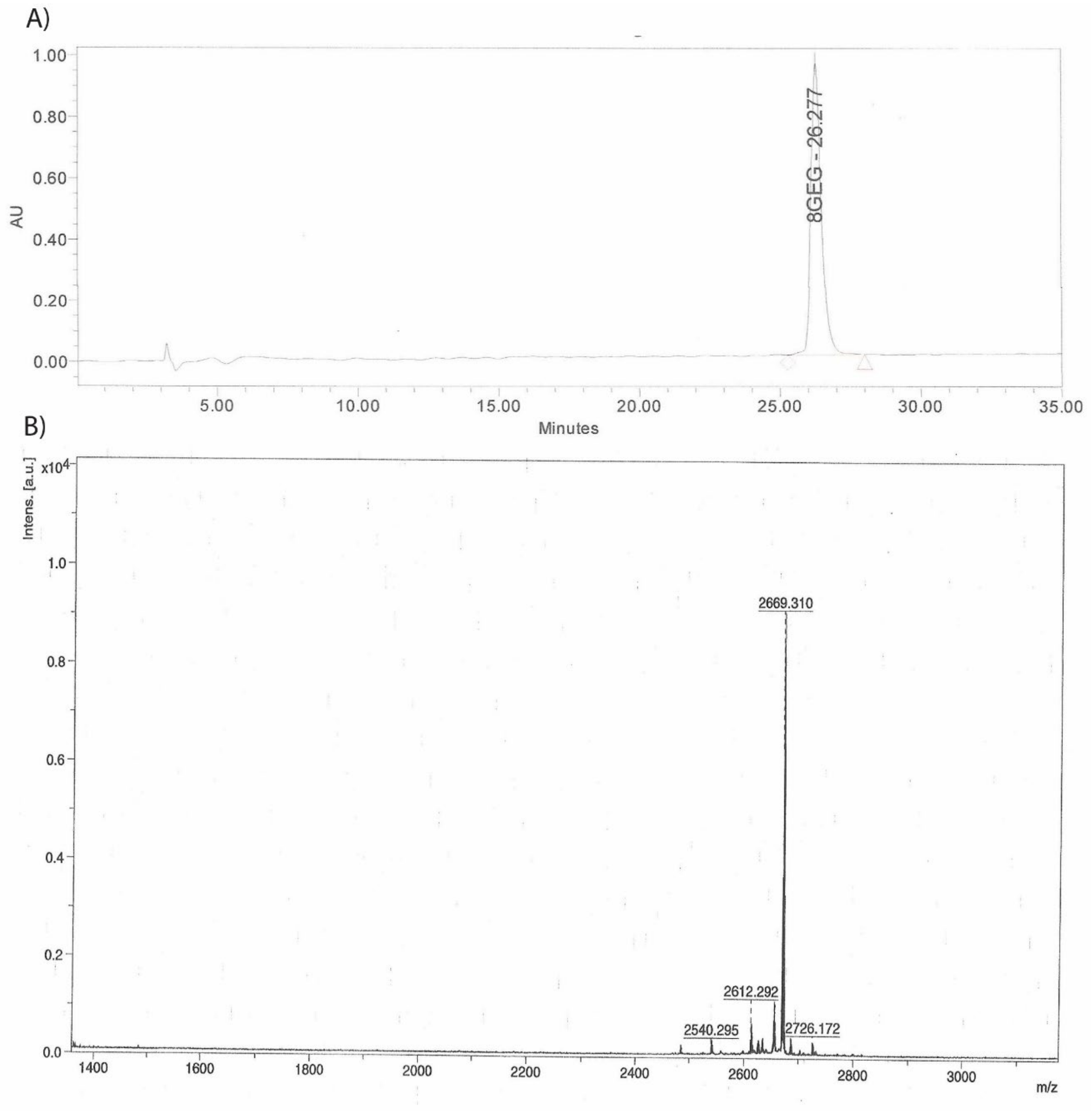

Figure S2 HPLC trace and mass spectrometry analysis (MALDI-TOF) of the H7 peptide. (A) HPLC separation was performed with a linear 5% to 55% gradient of solvent B (0.1% TFA in acetonitrile) into A (0.1% TFA in water) over 50 min at a 1 mL/min flow rate with UV detection at 442 nm. (B) MALDI-TOF of the peptide yielded an observed mass of 2777.580 Da ($[M+H]^+$)

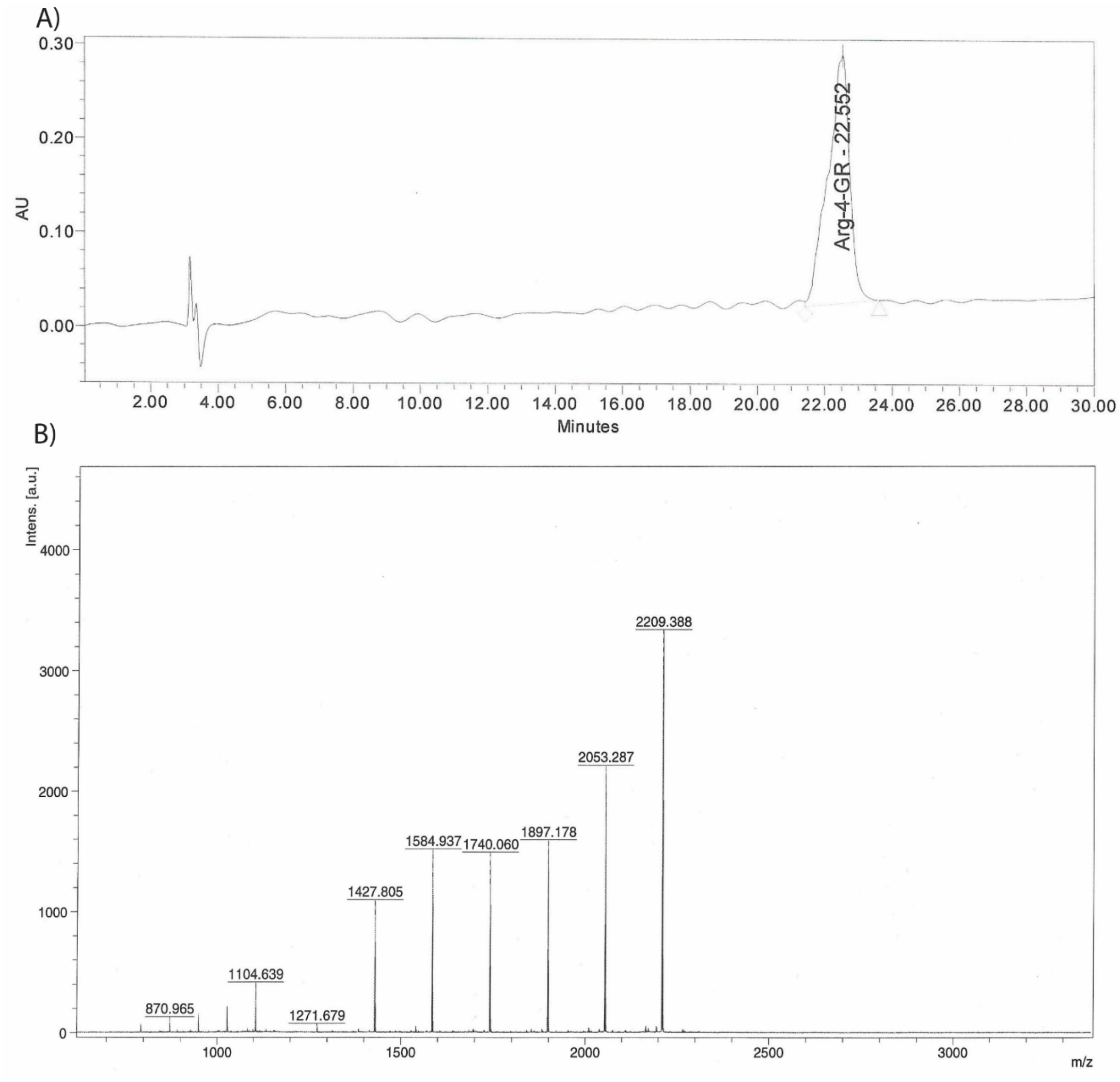

Figure S3. HPLC trace and mass spectrometry analysis (MALDI-TOF) of the R2 peptide. (A) HPLC separation was performed with a linear 5% to 55% gradient of solvent B (0.1% TFA in acetonitrile) into A (0.1% TFA in water) over 50 min at a 1 mL/min flow rate with UV detection at 442 nm. (B) MALDI-TOF of the peptide yielded an observed mass of 2777.580 Da ($[M+H]^+$)

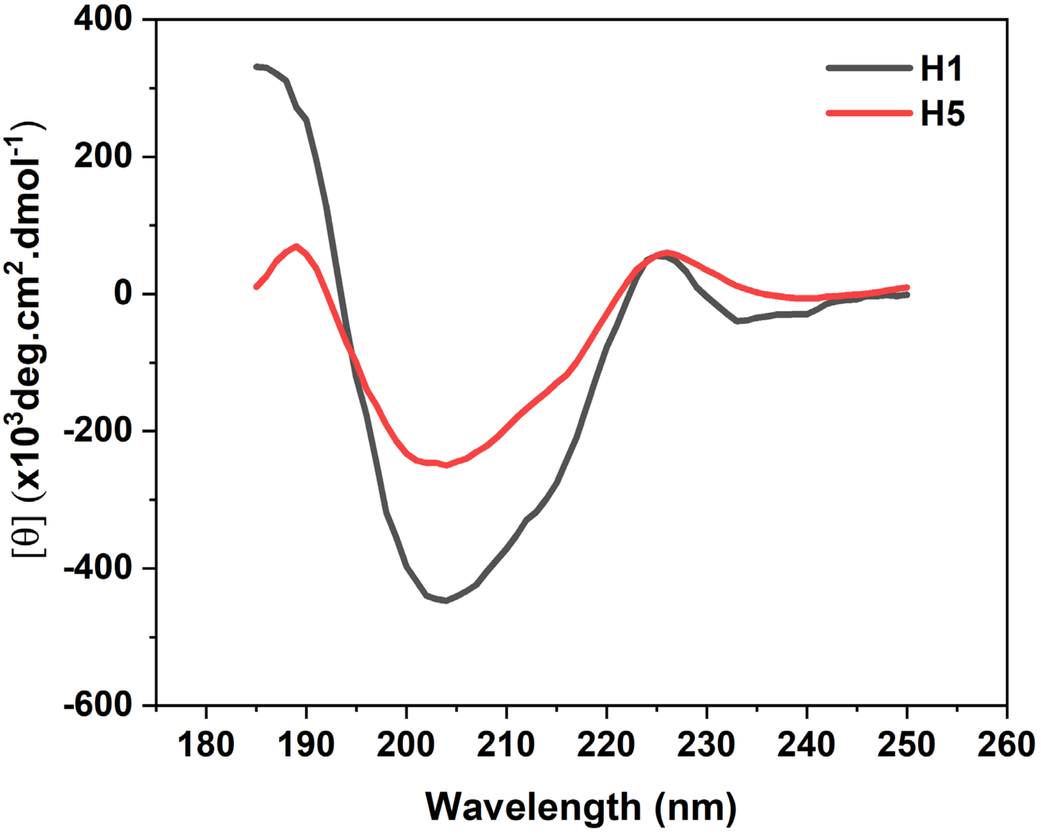

Figure S4. CD spectra of CPP/cargo complex. Experiments were performed using 40 µM peptide solution and 10 mM sodium phosphate buffer at 25°C. Both H1 and H5 exhibited spectra associated with a well-folded β-sheet confirmation with a minimum near 205 nm.

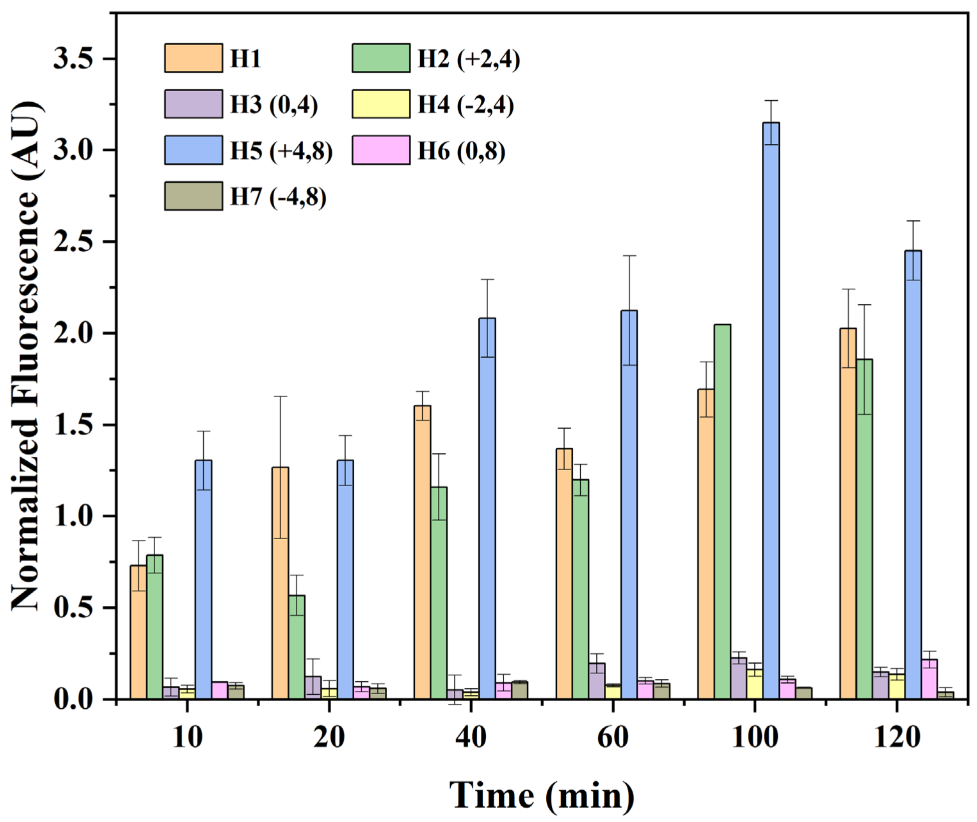

Figure S5. Structured CPP/cargo complexes with time-dependent internalization of peptides in HeLa cells. Intact cells were incubated with 10 µM of peptide solution for indicated times followed by lysis and quantification by fluorometry. All data are representative of triplicate experiments to produce the error bars. Results show increased uptake by complexes with positively charged cargoes and diminished uptake by complexes with neutral and negatively charged cargoes.

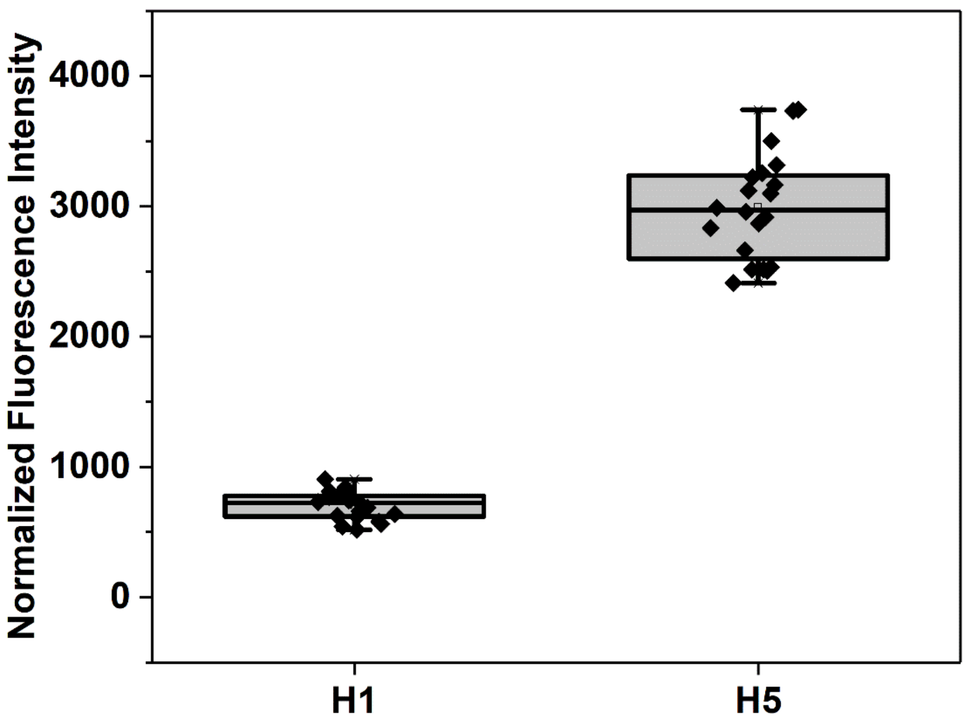

Figure S6. Comparison of fluorescent intensity of internalized peptides in HeLa cells. A) Line scans across 20 single cells were recorded and normalized against background signal to compare the average intensity of fluorescent signals for internalized peptides. The results demonstrate a higher normalized fluorescent intensity from the internalization of H5 compared to H1, in line with fluorometry data reported in Figure 1A.

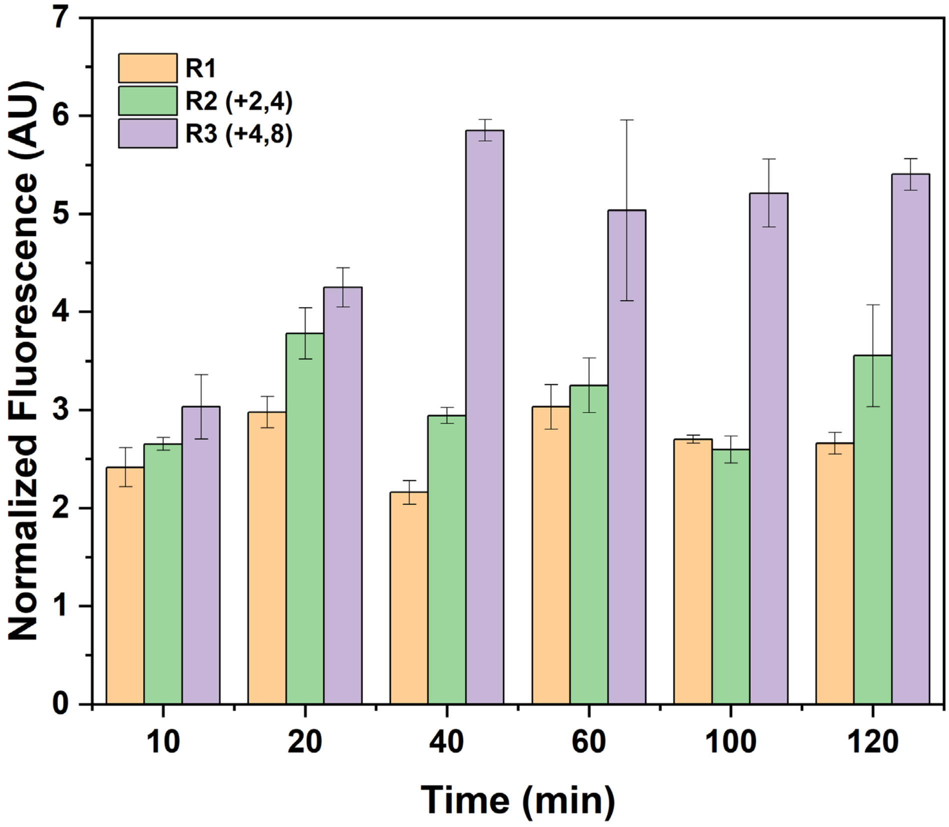

Figure S7. Unstructured CPP/cargo complexes with time-dependent internalization of peptides in HeLa cells. Intact cells were incubated with 10 μM of peptide solution for indicated times followed by lysis and quantification by fluorometry. All data are representative of triplicate experiments to produce the error bars. Results show increased uptake by complexes with positively charged cargoes.

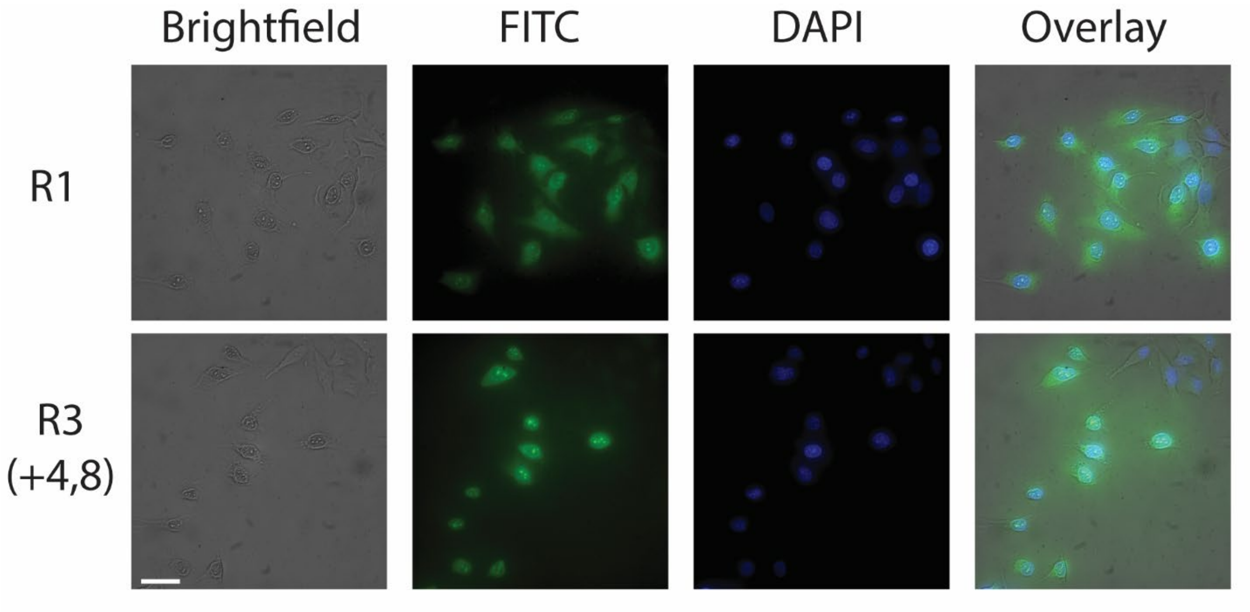

Figure S8. Visualization of intracellular distribution of unstructured CPP/cargo complexes in HeLa cells. A total of 10 µM peptide solutions were incubated with cells seeded on glass imaging chambers for 60 minutes at 37°C. Cells were washed with ECB and incubated with 8 µM Hoechst stain for 20 minutes prior to imaging. Representative images include brightfield, FITC (for peptide uptake), and DAPI (Hoechst) filters. Scale bar is 50 µm. Images demonstrate increased uptake with the addition of positive residues in the cargo (R3) compared to the CPP R1

Table S1. One-way ANOVA of the effect of time on CPP/cargo complex uptake. A single factor ANOVA was run on each peptide tested with the null hypothesis: $\mu10 = \mu20 = \mu40 = \mu60 = \mu100 = \mu120$. '$\mu$' denotes mean normalized fluorometry signal reported in Figure S2 (e.g., there is no significant effect of time on the mean signals for different experimental conditions). The data demonstrates that peptides H1, H2, H4, H5, and H6 demonstrate time dependence.

Peptide	*F(5,12)*	*P value*
H1	5.72	0.006
H2	33.40	<0.0001
H3	2.52	0.088
H4	5.77	0.006
H5	27.29	<0.0001
H6	5.89	0.006
H7	0.90	0.513

Table S2. Statistical comparison of net cargo charge on CPP/cargo complex uptake. The average fluorescent signals were compared for varying cargo charges using a Fisher LSD. The results showed that an increase in net cargo charge led to a significant increase in peptide internalization.

	**MeanDiff**	**SEM**	**t Value**	**P value**
H7 vs. H1	-0.743	0.091	-8.197	<0.0001
H4 vs. H1	-0.782	0.091	-8.624	<0.0001
H4 vs. H7	-0.039	0.091	-0.426	0.676
H6 vs. H1	-0.758	0.091	-8.359	<0.0001
H6 vs. H7	-0.015	0.091	-0.162	0.874
H6 vs. H4	0.024	0.091	0.265	0.795
H2 vs. H1	0.697	0.091	7.686	<0.0001
H2 vs. H7	1.440	0.091	15.883	<0.0001
H2 vs. H4	1.479	0.091	16.310	<0.0001
H2 vs. H6	1.455	0.091	16.045	<0.0001
H5 vs. H1	1.685	0.091	18.582	<0.0001
H5 vs. H7	2.428	0.091	26.779	<0.0001
H5 vs. H4	2.467	0.091	27.205	<0.0001
H5 vs. H6	2.443	0.091	26.941	<0.0001
H5 vs. H2	0.988	0.091	10.895	<0.0001
H3 vs. H1	-0.627	0.091	-6.918	<0.0001
H3 vs. H7	0.116	0.091	1.279	0.222
H3 vs. H4	0.155	0.091	1.706	0.110
H3 vs. H6	0.131	0.091	1.441	0.172
H3 vs. H2	-1.324	0.091	-14.604	<0.0001
H3 vs. H5	-2.312	0.091	-25.500	<0.0001

Table S3. One-way ANOVA on of the effect of net cargo length on the internalization of CPP/cargo complexes. A single factor ANOVA was run with the null hypothesis: $\mu H3 = \mu H8 = \mu H9$. 'μ' denotes mean normalized fluorometry signal reported in Figure 1B (e.g., there is no significant effect of peptide cargo length on the mean signals for different experimental conditions). The results demonstrate that net cargo length has no significant effect on the mean signals.

F(2,6)	*P value*
1.88	0.23

Table S4. One-way ANOVA of the effect of CPP/cargo complex uptake on HeLa cell viability. A single factor ANOVA was run on each concentration of peptide tested with the null hypothesis: $\mu H1 = \mu H2 = \mu H5$. 'μ' denotes mean normalized fluorometry signal reported in Figure 2 (e.g., there is no significant effect of peptide cargo on the mean signals for different experimental conditions). The data demonstrates that the addition of peptide cargo has no significant effect on the mean signals of under the four specified experimental conditions.

**Concentration**	*F(2,6)*	*P value*
5 μM	0.08	0.92
10 μM	0.38	0.70
20 μM	1.76	0.25
30 μM	1.19	0.37

Table S5. Statistical comparison of net cargo charge on CPP/cargo complexes using both structured (H) and unstructured (R) CPPs. The average fluorescent signals were compared for varying cargo charges using a Fisher LSD. The results showed an increase in the charge of cargoes conjugated to poly-arginine resulted in statistically significant differences in peptide internalization. Additionally, cargoes conjugated to poly-arginine demonstrated a statistically significant difference in uptake compared to the corresponding cargo conjugated to the RWRWR CPP.

	MeanDiff	SEM	t Value	P value
R4 vs. H9	1.397	0.173	8.096	<0.0001
H1 vs. H9	0.758	0.173	4.392	<0.0001
H1 vs. R4	-0.639	0.173	-3.704	0.00193
R1 vs. H9	2.950	0.173	17.094	<0.0001
R1 vs. R4	1.553	0.173	8.998	<0.0001
R1 vs. H1	2.192	0.173	12.702	<0.0001
H2 vs. H9	1.455	0.173	8.430	<0.0001
H2 vs. R4	0.058	0.173	0.334	0.74263
H2 vs. H1	0.697	0.173	4.038	<0.0001
H2 vs. R1	-1.495	0.173	-8.664	<0.0001
R2 vs. H9	3.882	0.173	22.490	<0.0001
R2 vs. R4	2.484	0.173	14.394	<0.0001
R2 vs. H1	3.124	0.173	18.098	<0.0001
R2 vs. R1	0.931	0.173	5.396	<0.0001
R2 vs. H2	2.427	0.173	14.060	<0.0001
H5 vs. H9	2.443	0.173	14.155	<0.0001
H5 vs. R4	1.046	0.173	6.059	<0.0001
H5 vs. H1	1.685	0.173	9.763	<0.0001
H5 vs. R1	-0.507	0.173	-2.939	0.00962
H5 vs. H2	0.988	0.173	5.724	<0.0001
H5 vs. R2	-1.439	0.173	-8.336	<0.0001
R3 vs. H9	6.236	0.173	36.131	<0.0001
R3 vs. R4	4.839	0.173	28.035	<0.0001
R3 vs. H1	5.478	0.173	31.739	<0.0001
R3 vs. R1	3.286	0.173	19.037	<0.0001
R3 vs. H2	4.781	0.173	27.701	<0.0001
R3 vs. R2	2.354	0.173	13.641	<0.0001
R3 vs. H5	3.793	0.173	21.976	<0.0001

Table S6. One-way ANOVA of the effect of time on unstructured CPP/cargo complex uptake. A single factor ANOVA was run on each peptide tested with the null hypothesis: $\mu 10 = \mu 20 = \mu 40 = \mu 60 = \mu 100 = \mu 120$. 'μ' denotes mean normalized fluorometry signal reported in Figure S7 (e.g., there is no significant effect of time on the mean signals for different experimental conditions). The data shows that peptides R2 and R3 demonstrate time dependence.

**Peptide**	***F(5,12)***	***P value***
R1	1.72	0.205
R2	6.25	0.005
R3	3.46	0.036
